# Focal Autonomic Seizures Manifesting With Prevailing Signs of Gastrointestinal Disorder in Dogs

**DOI:** 10.1111/jvim.70158

**Published:** 2025-06-11

**Authors:** Solene Diop, Elsa Lyon, Nicolas Van Caenegem, Catherine Escriou, Valérie Freiche, Stéphane Blot

**Affiliations:** ^1^ Ecole Nationale Vétérinaire d'Alfort Maisons‐Alfort France; ^2^ Univ Paris Est Créteil, INSERM, U955 IMRB “Biology of the Neuromuscular System” Team Maisons‐Alfort France; ^3^ Small Animal Internal Medicine Department, Neurology Service Vet AgroSup Marcy l'Etoile France; ^4^ Clinique Vétérinaire AniCura TRIOVet Rennes France

**Keywords:** canine, digestive, electroencephalography, epilepsy, episodic, ptyalism

## Abstract

In human medicine, focal seizures can clinically express as autonomic signs, such as gastrointestinal dysfunction, cardiovascular changes, and variation of pupillary size; but little is known about possible presentations of autonomic seizures in veterinary medicine. Three dogs were presented for recurrent episodes characterized by hypersalivation, vomiting, retching, and signs of abdominal discomfort. Neurological examinations were normal between episodes. Electroencephalographic (EEG) recordings identified ictal or interictal epileptiform discharges in all dogs. Based on clinical signs and EEG findings, a diagnosis of focal autonomic seizures was made. There was a notable positive response of clinical signs to antiepileptic treatment. These cases highlight the diverse clinical presentations of focal autonomic seizures in dogs and emphasize the diagnostic value of EEG in these cases.

**Abbreviations**
EEG: electroencephalography
MTLe: mesial temporal lobe epilepsy
SeLEAS: self‐limiting epilepsy with autonomic seizures

## 1 Introduction

As defined by the International Epilepsy Task Force in 2015, a seizure is “a transient occurrence of signs due to abnormal excessive or synchronous neuronal activity in the brain” [1]. Seizures can present with a diverse array of clinical manifestations, posing a key challenge for neurologists in discerning if the paroxysmal event is truly of epileptic nature [2].

Autonomic seizure is defined as “a seizure characterized by altered autonomic function of any type at seizure onset or in which manifestations consistent with altered autonomic function are prominent (quantitatively dominant or clinically important) even if not present at seizure onset” [3]. In human medicine, various types of autonomic seizures have been described, encompassing signs of gastrointestinal disorder (such as retching, ptyalism, nausea, vomiting, abdominal discomfort, borborygmia, diarrhea), alterations in cardiovascular function (bradycardia, tachycardia), fluctuations in pupillary size (mydriasis and miosis), and other clinical signs [4]. Diagnosing this type of seizure is known to be particularly challenging, because signs are commonly either disregarded as unrelated to seizures or inaccurately attributed to conditions such as migraine, syncope, or gastroenteritis [3, 5]. Several epileptic syndromes are described in human literature, including self‐limiting epilepsy with autonomic seizures or SeLEAS (formerly known as Panayiotopoulos syndrome), Rolandic epilepsy in children, and mesial temporal lobe epilepsy in adults [4]. Because of the possible confusion with other non‐epileptic conditions, electroencephalography (EEG) is considered the gold standard method of diagnosis. While interictal EEG in patients with autonomic seizures can be normal, there can be epileptiform changes or intermittent slowing observed in the anterior temporal EEG derivations, which become more pronounced during sleep [6, 7].

In veterinary medicine, little information is available regarding autonomic seizures. Phenobarbital‐responsive sialadenosis in dogs could represent an epileptic syndrome [8, 9, 10]. This hypothesis was supported by the observation of epileptiform waves on EEG recordings during episodes in one of the dogs under investigation. However, there is limited knowledge regarding whether autonomic seizures can manifest as other clinical signs. To provide a more detailed characterization of autonomic seizures in dogs, we present three cases in which seizures primarily feature autonomic signs, specifically involving disturbances in the gastrointestinal system.

## 2 Materials and Methods

Video‐EEG recordings were made using a wired EEG device (Brainbox 1042 Braintronics BV, Fl, the Netherlands) with EEG software (Coherence 7.1.3.2037 Natus Europe GMBH, Planegg, Germany). The recordings were carried out by one of the authors, E. Lyon. The montage used was bipolar, with surface cup electrodes. The placement of the eight electrodes is described in the reference [11] study. Both case no. 2 and no. 3 were conscious and unsedated during recording. Case no. 1 was administered a continuous infusion of midazolam at a rate of 0.3 mg/kg/h for the management of nonconvulsive status epilepticus, along with antiseizure medications such as phenobarbital and levetiracetam. Details are provided in Data S1.

### 2.1 Case Presentation

Description of clinical signs of all three cases is summarized in Table S1.

#### 2.1.1 Case No. 1

A 3‐year‐old male intact Jack Russell Terrier dog was presented to the internal medicine consultation at Ecole nationale vétérinaire d'Alfort (ENVA) for spastic regurgitations, ptyalism, and dysorexia with weight loss (20%) lasting for 3 weeks. After an episode of dietary indiscretion, the dog presented acute regurgitations of both alimentary and non‐alimentary content occurring several times a day, accompanied by nearly constant ptyalism. The exact frequency of episodes was hard to determine, but it was roughly estimated to be between 20 and 25 episodes per hour. The dog also exhibited non‐alimentary vomiting. Clinical signs did not improve with symptomatic treatments such as metoclopramide, maropitant, and omeprazole.

The dog was presented with a clinical dehydration level of 7%, was lethargic, and exhibited mild enlargement of the parotid glands. The dog displayed discomfort during abdominal palpation, and manipulations of the dog triggered several episodes of regurgitation. The neurological examination was normal, except for regurgitations and constant ptyalism.

**FIGURE 1:**
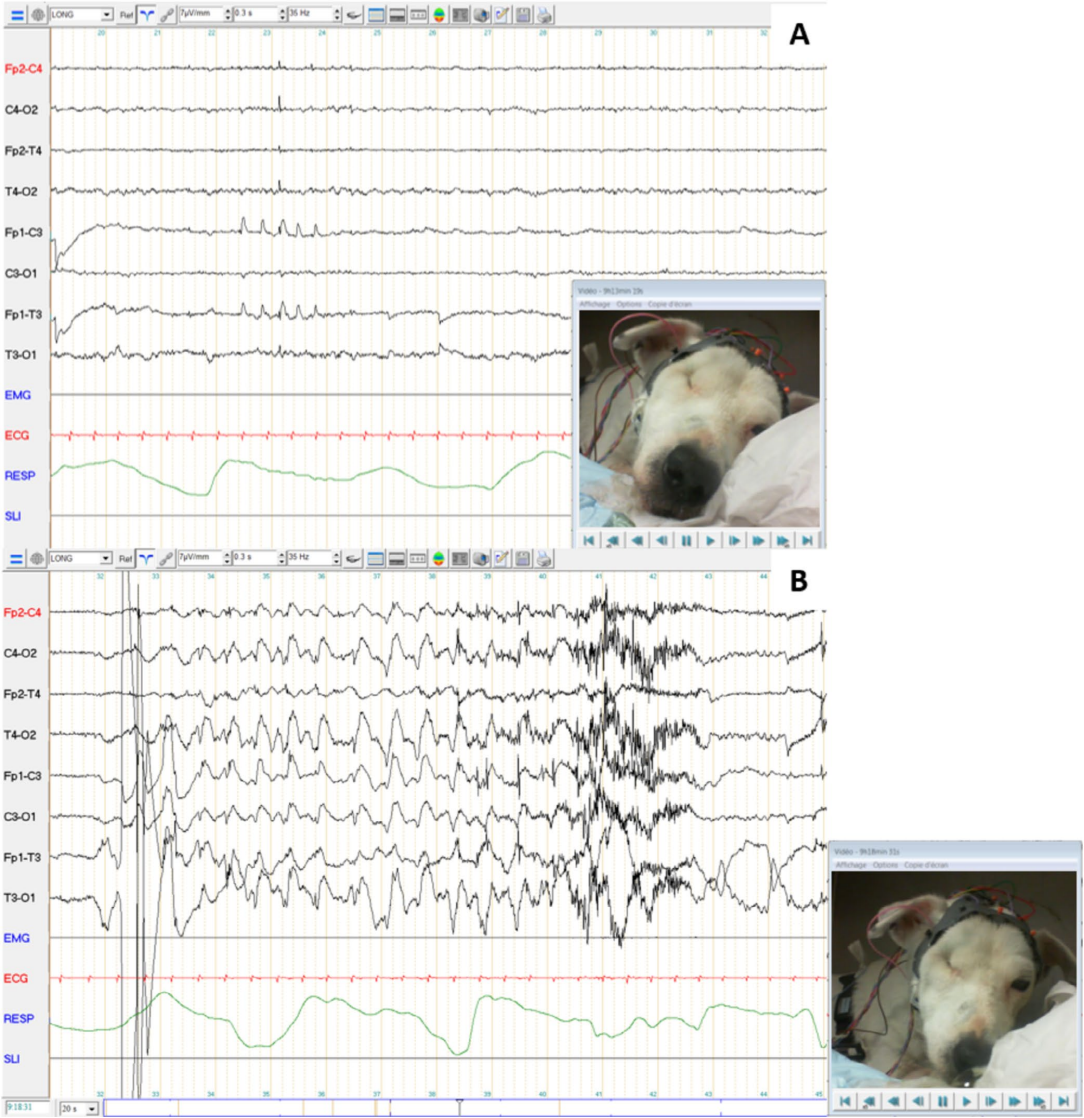
Electroencephalography recording of case no. 1. Symmetrical background activity was observed with low amplitude beta rhythm during wakefulness, and with medium‐amplitude theta and delta rhythm and slow waves during rest, without it being possible to differentiate between drowsiness and sleep stages (A). Bursts of spike‐and‐slow‐wave complexes in the left anterior region and isolated generalized spikes were observed without clinical signs (A) Paroxysmal manifestations with salivation, belching, and swallowing were observed 23 times during the 40‐min EEG recording. Generalized spikes mixed with movement and muscle artifacts were then observed (B).

Routine serum biochemistry, pre‐ and postprandial bile acids, canine pancreatic lipase, basal cortisol, and complete blood count results were within reference intervals (RI). Ionogram revealed marked hypokalemia (2.5 mmol/L; RI, 3.9–5.3), attributed to digestive potassium loss. To search for a radio‐opaque foreign body, abdominal and esophageal radiographs were conducted, yielding no notable findings. An abdominal ultrasound followed by an endoscopic screening of the upper gastrointestinal tracts was performed and did not reveal any abnormalities. The EEG was performed during 40 min when the dog was hospitalized for a quasi‐permanent salivation episode (Figure 1), suspected to be a nonconvulsive status epilepticus. Bursts of spike‐and‐slow‐wave complexes in the left anterior region and isolated generalized spikes were observed without clinical signs. Paroxysmal manifestations with salivation, belching, and swallowing were observed 23 times during the 40‐min EEG recording; during those episodes, generalized spikes mixed with movement and muscle artifacts were observed.

Phenobarbital was initiated with a loading dose of 15 mg/kg given over 24 h in three doses, then was followed by a dose of 3 mg/kg every 12 h. Due to the continuous regurgitations and vomiting, phenobarbital was initially administered intravenously. There was a gradual improvement in clinical signs during the hospitalization, 48 h after receiving phenobarbital. However, due to the persistence of intense regurgitations, the phenobarbital dosage was increased to 6 mg/kg every 12 h. On Day 12 after the initiation of treatment, the dog showed complete resolution of clinical signs.

The dog remained seizure free for 4 years. During that period, phenobarbital was continuously administered. Then, the dog presented a new episode of nearly constant regurgitations, vomiting, epigastric pain, abdominal distension, and diarrhea. A thorough investigation of gastrointestinal disease was conducted but failed to identify the underlying cause. Relapse of autonomic seizures was then suspected. Levetiracetam was added and progressively increased until a dose of 100 mg/kg/q8h PO. After initial improvement, seizure frequency and intensity worsened. Potassium bromide was added with a loading dose of 150 mg/kg/day for 3 days, then 25 mg/kg every 12 h. Clinical remission (absence of episodes) occurred 3 days after initiation of tritherapy. During the weeks after hospitalization, a gradual recurrence of seizures was observed. The addition of imepitoin at a dose of 30 mg/kg every 12 h had limited efficacy in controlling the seizures. The dog was euthanized 3 months after discharge due to persistent cluster episodes (exact frequency was not detailed in medical records). Necropsy was not performed.

#### 2.1.2 Case No. 2

A 7‐year‐old female spayed Podenco was presented to our hospital for episodes of gastrointestinal disorders occurring for 3 years. Approximately twice a week, the dog displayed clinical signs including ptyalism, coughing, retching, regurgitation, pica, epigastric pain, excessive repetitive swallowing movements, and borborygmi, lasting several hours. The postictal phase was identified by the owners and consisted of lethargy and intense fatigue. Otherwise, the dog exhibited normal behavior between episodes. Prior to the presentation, the frequency of seizures had increased, with a minimum of three episodes per day and a median of five episodes per day.

During consultation, the dog presented constant ptyalism and coughing. The rest of the physical examination was normal. Neurological examination was also normal.

**FIGURE 2:**
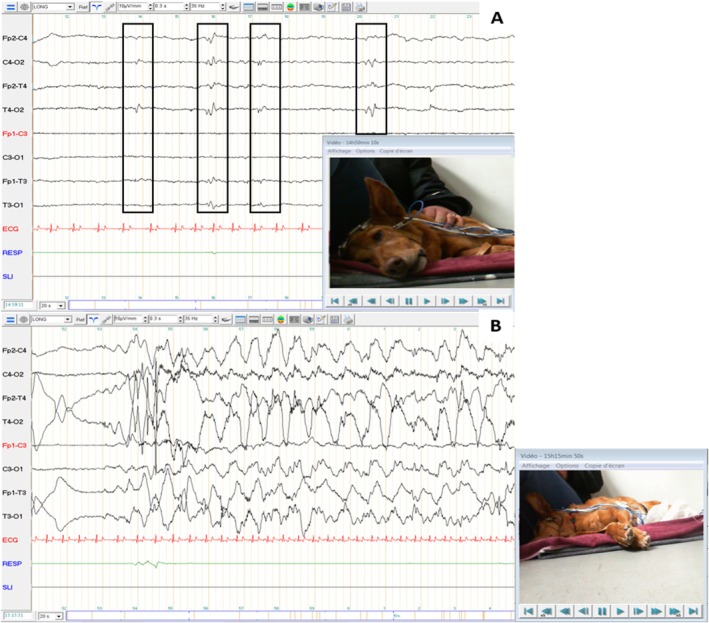
Electroencephalography recording of case no. 2. Symmetrical background activity was observed with low amplitude beta rhythm during wakefulness, and with medium‐amplitude theta rhythm during drowsiness (A). Isolated occipital spikes (boxes) were observed without clinical signs (A). No abnormalities were observed during the intermittent photic stimulation procedure. During drowsiness, rhythmic swallowing movements were suddenly observed. On the EEG, rhythmic spike‐and‐slow‐wave complexes superimposed on swallowing movement artifacts were observed, with a sudden onset and offset and with an increased heart rate (B).

Routine serum biochemistry, pre and postprandial bile acids, ionogram, and complete blood count were within reference intervals. Abdominal ultrasound and endoscopy of the upper abdominal tracts revealed no abnormalities. Histology of the endoscopic biopsy showed mild chronic superficial lymphoplasmacytic gastritis and duodenitis, which were deemed insufficient to account for the observed clinical signs. Thoracic radiographs confirmed the absence of megaesophagus and ruled out intrathoracic causes for the cough. Echocardiography findings were similar to the examination conducted during the previous year. Magnetic resonance imaging (MRI) of the brain yielded no notable findings, and the cerebrospinal fluid (CSF) analysis yielded no abnormalities. An EEG was recorded for 40 min during natural wakefulness and drowsiness, with an intermittent photic stimulation procedure at the beginning of the session (Figure 2). Isolated occipital spikes (boxes) were observed without clinical signs. During drowsiness, rhythmic swallowing movements were suddenly observed. On the EEG, rhythmic spike‐and‐slow‐wave complexes superimposed on swallowing movement artifacts were observed, with a sudden onset and offset and with an increased heart rate.

Phenobarbital was started orally at a dose of 3.3 mg/kg twice daily. After initiation, the seizure frequency decreased to maximum of two seizures per day. Doses were gradually increased to 4.5 mg/kg twice daily. Potassium bromide was added with a loading dose of 600 mg/kg over 5 days and maintained at 40 mg/kg/day. Insufficient response prompted a gradual increase in bromide dosage, reaching 60 mg/kg/day. Higher doses led to pronounced sedation and ataxia. Seizure frequency decreased to two seizures over 3‐day period. Persistent ataxia and lethargy led to a decreased potassium bromide dosage to 28 mg/kg/day and levetiracetam was added at 28 mg/kg every 8 h. Subsequently, the seizure frequency decreased to about one seizure every 2–3 days for a year and 9 months (with a maximum range of 7 days between seizures).

Twenty‐one months after the initiation of phenobarbital, the dog exhibited cluster generalized tonic–clonic seizures. The preictal phase was characterized as hyperexcitation, disorientation, hyperesthesia, and facial pruritus lasting about 60 s. Intense fatigue was identified between episodes. He was readmitted to the hospital, where he was additionally diagnosed with aspiration pneumonia.

After loading dose, the bromide dosage was increased and there were no further occurrences of tonic–clonic seizures. Signs of respiratory disease worsened over a period of 5 days, and the dog died due to cardiorespiratory arrest during hospitalization. Necropsy was not performed.

#### 2.1.3 Case No. 3

**FIGURE 3:**
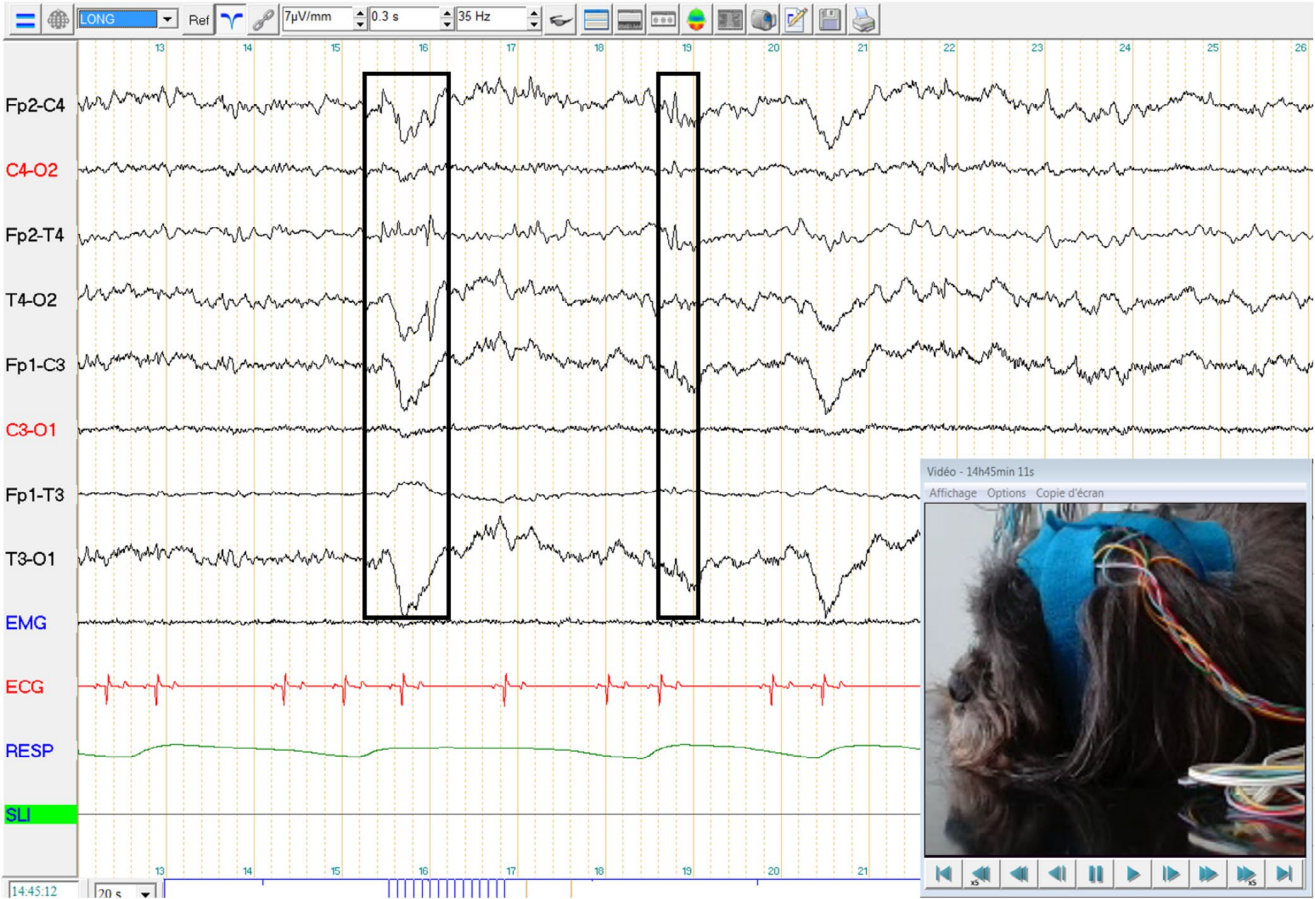
Electroencephalography recording of dog no. 3. Symmetrical background activity was observed with low amplitude beta and alpha rhythms during wakefulness, and with medium‐amplitude theta rhythm during drowsiness. Frontotemporal isolated spikes (boxes) and polyspikes of moderate amplitude were observed during drowsiness. No abnormalities were observed during the intermittent photic stimulation procedure.

A 5‐year‐old intact male Lhasa Apso was presented to the gastroenterology consultation for paroxysmal gastrointestinal episodes (characterized by rumbling, nausea, ptyalism, vomiting without food content, and epigastric pain) lasting several hours, since his adoption 3 years before presentation. The dog also showed cluster episodes of impaired awareness, chewing, and licking his paws since adoption, especially during rest. Initial frequency of seizures was about 10–12 episodes of gastrointestinal disease per month, along with 8–10 episodes of stereotypical behavior per month. Physical examination and neurological examination were normal. Routine serum biochemistry, bile acids dosage, basal cortisol, and canine lipase were within reference intervals. The abdominal ultrasound did not reveal significant abnormalities. Mild signs of gastritis were present on endoscopic examination. Endoscopic biopsies of upper and lower digestive tracts were consistent with mild lymphoplasmocytic gastroenteritis. Those findings were insufficient to explain clinical signs, and poor response to treatment of chronic gastroenteritis was observed. The EEG was performed the day after an episode of digestive discomfort. In addition, the owners reported episodes of myoclonus when falling asleep, encouraged by physical exercise. So, the EEG was performed after a long walk. EEG (Figure 3) was performed for 40 min and included an intermittent photic stimulation procedure. Fronto‐temporal isolated spikes (boxes) and polyspikes of moderate amplitude were observed during drowsiness.

After initiation of levetiracetam therapy at 20 mg/kg every 8 h, an 80% decrease in seizure frequency was noted (with 0–2 episodes per month). Additionally, owners reported diminished severity in the clinical presentation (episodes were less intense) and a reduction in their duration (less than an hour).

Six months after initiation of levetiracetam, there was an increase in seizure frequency (4–6 episodes per month), necessitating adjusting the levetiracetam dosage (93 mg/kg every 8 h) and introducing phenobarbital (3.1 mg/kg every 12 h). The dog stabilized with 0–2 episodes per month, with diminished severity (one vomiting per episode, mild restlessness, licking and chewing his paws) and reduced duration (usually less than 1 h).

Owners have consistently rated the dog's quality of life as very good since the initiation of antiepileptic therapy. The dog was still alive at the time of writing, after 2 years of follow‐up.

## 3 Discussion

This case series highlights the broad range of clinical manifestations observed in focal autonomic seizures, a diversity similarly noted in human medicine. Clinical signs in our study differed from previously reported cases of phenobarbital‐responsive sialadenosis. Only one dog showed enlarged salivary glands, despite all dogs exhibiting ictal ptyalism.

During paroxysmal events, the presence of pre‐ or postictal phases, altered consciousness, and/or autonomic signs supports the classification of the event as a seizure, as outlined by the International Veterinary Epilepsy Task Force [2]. However, in focal autonomic seizures, pre‐ and postictal phases are not necessarily present. Notably, only one dog in our case series exhibited a postictal phase. Because of the large array of presentations, focal autonomic seizures should be considered in dogs exhibiting recurrent paroxysmal episodes of digestive disorders that had extensive digestive work‐ups that failed to identify a causative disease, and especially when there is a poor response to chronic gastroenteritis treatment.

Because of the confounding aspect of seizure, other elements can confirm the epileptic nature of the episodes. In particular, the presence of other types of seizure might be useful. In our study, one dog experienced generalized tonic–clonic seizures in addition to focal autonomic seizures. Another dog exhibited behavioral seizures with impaired awareness, chewing and licking his paws. However, the last dog only presented one type of seizure, and thus its absence should not definitively rule out the epileptic nature of the episodes.

The duration of autonomic seizure is unusual. Typically, seizures have a duration of less than 5 min, or they are classified as status epilepticus. In human medicine, focal simple seizures typically have a shorter duration compared to generalized tonic–clonic seizures [12]. However, focal status epilepticus is considered common in certain syndromes, such as SeLEAS (44% of seizures are more than 10 min in this syndrome, and frequently persists for hours). In our case series, all dogs exhibited a long duration of clinical signs, often for hours. When left untreated, epilepsy can be progressive, carrying risks of structural damage to the brain and occurrence of status epilepticus or cluster seizures [13, 14]. In these dogs, epilepsy might have progressed due to the long period of medical misdiagnosis before presentation.

Electroencephalography is an essential diagnostic tool to distinguish epileptic from non‐epileptic disorders, especially in autonomic seizures. In SeLAES, ictal and interictal EEG has showed high sensitivity. Ictal abnormal findings can be identified in up to 90% of patients [15]. Because there is a suspicion that EEG abnormalities are more likely to manifest during sleep studies, it is advisable that a sleep EEG recording should always be conducted if a routine EEG yields normal results [16]. Otherwise, several techniques can maximize the sensitivity of scalp EEG in temporal lobe epilepsy, including nasopharyngeal, sphenoidal, anterior temporal, and mandibular notch electrodes [17]. In many cases, when scalp EEG is inefficient, intracranial EEG is necessary to directly record activity from the brain parenchyma [18]; although this technique is not widely available in veterinary medicine. Prolonged video‐EEG monitoring increases the likelihood of recording spontaneous seizures, with a minimum of 24 h considered the gold standard [19, 20]. Recent literature is available on the clinical application of unanesthetized video‐EEG in veterinary medicine [2]. In these studies, EEG proved itself a powerful tool in identifying seizures in dogs [20, 21, 22]. In our study, we concluded that dogs experienced seizures due to the presence of EEG abnormalities during the ictal or interictal phase, and, at the very least, an initial response to treatment (a reduction of more than 50% in seizure frequency). Other diagnostic approaches exist for detecting seizures in dogs, including heart rate variability (HRV). It is interesting to note that one dog in our study displayed tachycardia during autonomic seizures (case n°2). This variable could be useful in detecting autonomic seizures if alteration in cardiovascular function is associated [23].

We suspected that the seizures, given that the dogs initially exhibited clinical signs between 6 months and 6 years of age, and had normal interictal neurological examinations, were indicative of idiopathic epilepsy. All dogs had a minimum follow up of 21 months, and did not develop interictal signs of neurological disease, supporting the diagnosis of idiopathic epilepsy. In this series, only one case had MRI of the brain and CSF analysis that excluded structural disease. However, no postmortem examination was performed to confirm the presumptive diagnosis of idiopathic epilepsy.

Given the suspicion of idiopathic epilepsy in all dogs, coupled with the presentation of recurrent cluster seizures or status epilepticus, phenobarbital was considered the preferred treatment over imepitoin or levetiracetam (according to International Epilepsy Task Force Consensus) [24]. However, levetiracetam was selected in one case due to its infrequent side effects. In previous descriptions of focal autonomic seizures, clinical signs responded positively to phenobarbital [8, 9, 10]. In our study, all dogs still presented frequent seizures after treatment. Further studies are needed to evaluate the efficiency of treatment for this type of seizure.

This case series highlights the variety of clinical manifestations observed in focal autonomic seizures in dogs. Dogs with such clinical signs are usually referred or presented for an internal medicine consultation. Diagnosis is often difficult and relies on a wide diagnostic workup. Clinical presentation, EEG recording, and treatment response are essential to characterize the disease, after exclusion of any gastrointestinal disease. Focal autonomic seizures should be considered in dogs exhibiting recurrent paroxysmal episodes of digestive disorders, along with no to moderate organic lesions, especially when there is a poor response to chronic gastroenteritis treatments.

## Disclosure

Authors declare no off‐label use of antimicrobials.

## Ethics Statement

Authors declare no institutional animal care and use committee or other approval was needed. Authors declare human ethics approval was not needed.

## Conflicts of Interest

The authors declare no conflicts of interest.

## Supporting information


**Data S1.** EEG protocol.

**Table S1.** Details on clinical presentation of three dogs with focal autonomic seizures.

## References

[1] R. S. Fisher , J. H. Cross , J. A. French , et al., “Operational Classification of Seizure Types by the International League Against Epilepsy: Position Paper of the ILAE Commission for Classification and Terminology,” Epilepsia 58 (2017): 522–530. 28276060 10.1111/epi.13670

[2] L. De Risio , S. Bhatti , K. Muñana , et al., “International Veterinary Epilepsy Task Force Consensus Proposal: Diagnostic Approach to Epilepsy in Dogs,” BMC Veterinary Research 11 (2015): 148. 26316175 10.1186/s12917-015-0462-1 PMC4552251

[3] C. D. Ferrie , R. Caraballo , A. Covanis , et al., “Autonomic Status Epilepticus in Panayiotopoulos Syndrome and Other Childhood and Adult Epilepsies: A Consensus View,” Epilepsia 48 (2007): 1165–1172. 17442005 10.1111/j.1528-1167.2007.01087.x

[4] B. Moseley , L. Bateman , J. J. Millichap , E. Wirrell , and C. P. Panayiotopoulos , “Autonomic Epileptic Seizures, Autonomic Effects of Seizures, and SUDEP,” Epilepsy & Behavior 26 (2013): 375–385. 23099286 10.1016/j.yebeh.2012.08.020

[5] C. Ferrie , R. Caraballo , A. Covanis , et al., “Panayiotopoulos Syndrome: A Consensus View,” Developmental Medicine and Child Neurology 48 (2006): 236–240. 16483404 10.1017/S0012162206000508

[6] J. A. French , P. D. Williamson , V. M. Thadani , et al., “Characteristics of Medial Temporal Lobe Epilepsy: I. Results of History and Physical Examination,” Annals of Neurology 34 (1993): 774–780. 8250525 10.1002/ana.410340604

[7] M. Javidan , “Electroencephalography in Mesial Temporal Lobe Epilepsy: A Review,” Epilepsy Research and Treatment 2012 (2012): 1–17. 10.1155/2012/637430 PMC3420622 22957235

[8] J. Stonehewer , A. J. Mackin , S. Tasker , J. w. Simpson , and I. G. Mayhew , “Idiopathic Phenobarbital‐Responsive Hypersialosis in the Dog: An Unusual Form of Limbic Epilepsy?,” Journal of Small Animal Practice 41 (2000): 416–421. 11023129 10.1111/j.1748-5827.2000.tb03236.x

[9] H.‐K. Chae , J.‐H. Lee , M. C. Choi , W.‐J. Song , and H.‐Y. Youn , “Successful Treatment of a Dog With Phenobarbital‐Responsive Sialadenosis and an Oesophageal Stricture,” Veterinary Medicine and Science 7 (2021): 660–664. 33410603 10.1002/vms3.416 PMC8136939

[10] E. Alcoverro , M. D. Tabar , A. Lloret , X. Roura , J. Pastor , and M. Planellas , “Phenobarbital‐Responsive Sialadenosis in Dogs: Case Series,” Topics in Companion Animal Medicine 29 (2014): 109–112. 25813851 10.1053/j.tcam.2015.01.003

[11] E. Lyon , H. Pochat , S. Blot , et al., “Use of Video‐Electroencephalography as a First‐Line Examination in Veterinary Neurology: Development and Standardization of Electroencephalography in Unsedated Dogs and Cats,” Frontiers in Veterinary Science 11 (2024): 1326165. 38343449 10.3389/fvets.2024.1326165 PMC10853351

[12] J. Dobesberger , A. J. Ristić , G. Walser , et al., “Duration of Focal Complex, Secondarily Generalized Tonic‐Clonic, and Primarily Generalized Tonic‐Clonic Seizures—A Video‐EEG Analysis,” Epilepsy & Behavior 49 (2015): 111–117. 25935513 10.1016/j.yebeh.2015.03.023

[13] K. D. Laxer , E. Trinka , L. J. Hirsch , et al., “The Consequences of Refractory Epilepsy and Its Treatment,” Epilepsy & Behavior 37 (2014): 59–70. 24980390 10.1016/j.yebeh.2014.05.031

[14] M. R. Sperling , “The Consequences of Uncontrolled Epilepsy,” CNS Spectrums 9 (2004): 98–101. 14999166 10.1017/s1092852900008464

[15] C. P. Panayiotopoulos , “Autonomic Seizures and Autonomic Status Epilepticus Peculiar to Childhood: Diagnosis and Management,” Epilepsy & Behavior 5 (2004): 286–295. 15145296 10.1016/j.yebeh.2004.01.013

[16] A. Graziosi , N. Pellegrino , V. di Stefano , U. Raucci , A. Luchetti , and P. Parisi , “Misdiagnosis and Pitfalls in Panayiotopoulos Syndrome,” Epilepsy & Behavior 98 (2019): 124–128. 31369969 10.1016/j.yebeh.2019.07.016

[17] J. R. Ives , F. W. Drislane , S. C. Schachter , et al., “Comparison of Coronal Sphenoidal Versus Standard Anteroposterior Temporal Montage in the EEG Recording of Temporal Lobe Seizures,” Electroencephalography and Clinical Neurophysiology 98 (1996): 417–421. 8647045 10.1016/0013-4694(96)95124-x

[18] M. O. Baud , K. Schindler , and V. R. Rao , “Under‐Sampling in Epilepsy: Limitations of Conventional EEG,” Clinical Neurophysiology Practice 6 (2020): 41–49. 33532669 10.1016/j.cnp.2020.12.002 PMC7829106

[19] K. Timpte , U. Rosenkötter , P. Honrath , Y. Weber , S. Wolking , and J. Heckelmann , “Assessing 72 h vs. 24 h of Long‐Term Video‐EEG Monitoring to Confirm the Diagnosis of Epilepsy: A Retrospective Observational Study,” Frontiers in Neurology 14 (2023): 1281652. 37928154 10.3389/fneur.2023.1281652 PMC10622959

[20] E. Folkard , L. Niel , L. Gaitero , and F. M. K. James , “Tools and Techniques for Classifying Behaviours in Canine Epilepsy,” Frontiers in Veterinary Science 10 (2023): 1211515. 38026681 10.3389/fvets.2023.1211515 PMC10646580

[21] R. Poma , A. Ochi , and M. A. Cortez , “Absence Seizures With Myoclonic Features in a Juvenile Chihuahua Dog,” Epileptic Disorders 12 (2010): 138–141. 20483714 10.1684/epd.2010.0312

[22] E. Folkard , C. McKenna , G. Monteith , et al., “Feasibility of In‐Home Electroencephalographic and Actigraphy Recordings in Dogs,” Frontiers in Veterinary Science 10 (2023): 1240880. 38260190 10.3389/fvets.2023.1240880 PMC10800542

[23] J. Bongers , R. Gutierrez‐Quintana , and C. E. Stalin , “The Prospects of Non‐EEG Seizure Detection Devices in Dogs,” Frontiers in Veterinary Science 9 (2022): 896030. 35677934 10.3389/fvets.2022.896030 PMC9168902

[24] S. F. M. Bhatti , L. de Risio , K. Muñana , et al., “International Veterinary Epilepsy Task Force Consensus Proposal: Medical Treatment of Canine Epilepsy in Europe,” BMC Veterinary Research 11 (2015): 176. 26316233 10.1186/s12917-015-0464-z PMC4552371

