# Supplementary material for: Focal Autonomic Seizures Manifesting With Prevailing Signs of Gastrointestinal Disorder in Dogs
Source: J Vet Intern Med. 2025 Jun 11;39(4):e70158. doi: 10.1111/jvim.70158 (PMC12152640; doi:10.1111/jvim.70158)
Supplement: Supplementary file 1 — Data S1. EEG protocol. [file JVIM-39-e70158-s002.docx]

**Supplementary Data 1.**  **EEG protocol:**

All video-EEG were performed by one of the authors, E. Lyon, in the presence of the animal's owner (for case n°2 and 3) or a clinician (case n°1).

Both case n°2 and n°3 were conscious and unsedated during recording. Case n°1 was administered a continuous infusion of midazolam at a rate of 0.3 mg/kg/h for the management of non-convulsive status epilepticus, along with antiseizure medications such as phenobarbital and levetiracetam. He was described as conscious but with reduced responsiveness.

EEG recordings were made using a wired EEG device (Brainbox^®^ 1042 Braintronics BV, Fl. The Netherlands) with EEG software (Coherence^®^ 7.1.3.2037 Natus Europe GMBH, Planegg, Germany). Montage used for the three cases was bipolar. The acquisition settings were sampling frequency per channel 256 Hz, high pass filter 0.3 s, low pass filter 35 Hz, resolution 7 μV/mm, longitudinal and transverse montages. Wired cup Ag electrodes (NE-112A, Nihon Kohden^®^, Tokyo, Japan; [Figure 1B](https://pmc.ncbi.nlm.nih.gov/articles/PMC10853351/#F1)) were used, along with elastic straps perforated every 1.5 cm, in which the electrodes were inserted. The placement of the eight electrodes was based on previous published literature. Conductive paste and gel (Ten20^®^, Weaver and Company, Aurora, CO, USA, and SignaGel^®^ Parker Laboratories, INC. Fairfield, NJ, USA) were applied between the skin and the electrode. Physiological sensors, such as electrocardiography, electromyography, and respiratory sensor were also used during recording. Intermittent Photic Stimulation (IPS) was conducted at the start of the examination while the animal was lying down and awake. The lamp was placed at eye level, 30 cm from the animal, and the program followed a sequence that gradually increased the frequency of light flashes: 3–5–7–10–13–15–17–20–25–30–35–40–45–50 Hz, with each flash lasting 10 seconds and 5-second pauses between frequency changes. If the animal fell asleep during the photic stimulation, the test was repeated at the end of the EEG examination after the animal had woken up.

All recordings were examined by three reviewers in joint reading sessions to obtain a consensus on diagnosis.
