# Supplementary material for: Focal Autonomic Seizures Manifesting With Prevailing Signs of Gastrointestinal Disorder in Dogs
Source: J Vet Intern Med. 2025 Jun 11;39(4):e70158. doi: 10.1111/jvim.70158 (PMC12152640; doi:10.1111/jvim.70158)
Supplement: Supplementary file 2 — Table S1. Details on clinical presentation of three dogs with focal autonomic seizures. [file JVIM-39-e70158-s001.docx]

|  | Ictal signs | Length of episodes | Initial frequency of episodes | Other type of seizures | Pre or post ictal phase | Cluster seizures | Non convulsive status epilepticus |
| --- | --- | --- | --- | --- | --- | --- | --- |
| Case n°1 | Spastic regurgitations, ptyalism, vomiting | Exact length unknown (less than 5 minutes) | 20 to 25 episodes in an hour | Not reported | Not identified | Yes | Yes |
| Case n°2 | Ptyalism, coughing, retching, regurgitations, pica, epigastric pain, excessive repetitive swallowing movements, borborygmi, | 1 to 2 hours | 5 episodes daily | Yes, cluster generalized tonic clonic seizures | Post ictal phase: lethargy and intense fatigue (1 to 2 hours) | Yes | No |
| Case n°3 | Pyalism, nausea, vomiting without food content, borborygmi, epigastric pain | 1 to 3 hours | 8 to 10 episodes daily | Yes, focal behavioural seizures (impaired awareness, chewing, and licking his paws) | Not identified | Yes | No |

**Supplementary Table 1**: **Details on clinical presentation of three dogs with focal autonomic seizures.**
